# Aortoiliac graft‐enteric fistula presenting as gastrointestinal hemorrhage: A report on a complex case management

**DOI:** 10.1002/ccr3.7801

**Published:** 2023-08-15

**Authors:** Michael Azzopardi, Tom Wallace, Yazan S. Khaled

**Affiliations:** ^1^ Department of Academic Surgery, Leeds Institute of Medical Research St James's University Hospital Leeds UK; ^2^ Department of Vascular Surgery, Leeds Vascular Institute Leeds General Infirmary Leeds UK; ^3^ School of Medicine University of Leeds Leeds UK

**Keywords:** artery‐enteric fistula, gastrointestinal hemorrhage, graft explant, sigmoid cancer

## Abstract

**Key Clinical Message:**

Iliac artery‐enteric fistula is a rare cause of lower GI bleeding and can cause life‐threatening consequences. A high degree of clinical suspicion is needed in patients with previous aortic surgery to allow early multidisciplinary intervention.

**Abstract:**

This case study discusses the staged management of a 78‐year‐old patient presenting with life‐threatening lower gastrointestinal (GI) bleeding secondary to an aortoiliac graft‐enteric fistula (GEF) into the sigmoid colon on the background of an adenocarcinoma and diverticular disease. The patient had an aorto bi‐iliac synthetic dacron graft repair of an abdominal aortic aneurysm (AAA) some 20 years ago. Here, we present a case of successful endovascular treatment of massive hemorrhage as a bridge to definitive second‐stage dacron graft explant and autologous vein reconstruction with a simultaneous anterior resection.

## Graphical abstract

Computed tomography images of the right common iliac artery pseudoaneurysm.

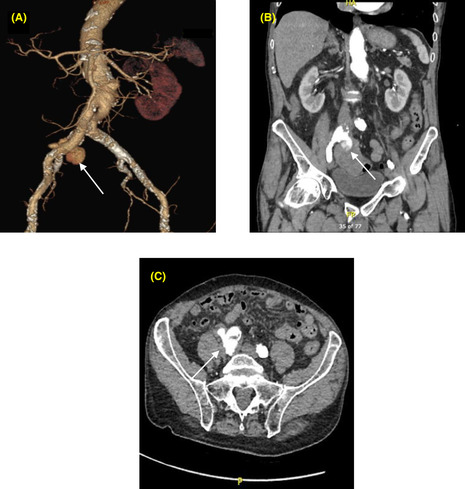

## 1 BACKGROUND

Arterio‐enteric fistula (ArEF) is an umbrella term that encompasses various fistulations between the great arteries and the gastrointestinal (GI) tract, including aortoesophageal, aortogastric, aorto‐enteric fistulas (AEF) and iliac artery‐enteric fistula (IEF).

Arterio‐enteric fistula is a rare cause of potentially lethal GI bleeding, and the vast majority appear to occur in the aorta with few examples of IEF encountered in modern literature. These fistulas can occur via two separate pathological mechanisms. Primary ArEFs are rare, occurring as a spontaneous communication between an artery and bowel from a combination of direct frictional forces and inflammatory processes.^1^ The most encountered mechanism contributing to primary ArEF is aneurysmal formation, which is theorized to develop into a fistula from the repetitive mechanical forces exacerbated by cardiac pulsations and peristaltic movements.^2^ This gradual degradation and erosion of the outermost layers of bowel and artery can also result spontaneously in other pathological circumstances, including tumors, diverticular disease, sepsis, syphilis, and tuberculosis.^3^, ^4^, ^5^, ^6^

Secondary ArEFs are much more common given that they are an iatrogenic complication of open or endovascular AAA repair using synthetic graft. In this case, seeding of bacteria onto the synthetic graft is known to exacerbate the erosive mechanisms involved in fistula formation.^2^ Hallet et al.^7^ have suggested a 1.6% chance of fistula formation after AAA graft repair. The vast majority of ArEFs encountered in literature are between the abdominal aorta and duodenum due to their intimate anatomical association. Iliac artery‐enteric fistulas are rarely encountered, but it appears that the majority occur secondary to pelvic surgery, malignancy, radiotherapy, and infection.^8^

In this case report, we encountered a 78‐year‐old patient, 20 years after an elective AAA Y‐graft repair, presenting to emergency with hematochezia as a result of a fistula between the right common iliac artery (CIA) and sigmoid colon. The patient was known to have a sigmoid adenocarcinoma and diverticular disease from a colonoscopy 3 weeks prior to his emergency presentation. The rarity and complexity of this case necessitated a multidisciplinary staged approach to the management, both in the acute and elective settings, leading to a favorable outcome.

## 2 CASE PRESENTATION

A 78‐year‐old male with a past medical history of atrial fibrillation (AF), psoriasis, hypertension, and hyperlipidemia presented to the emergency department with syncope after passing 500 mL of brisk fresh blood per rectum. Twenty years ago, he had an uncomplicated elective infrarenal aorto bi‐iliac graft repair of an AAA. His regular medications included bisoprolol, rivaroxaban, and methotrexate at the time of admission.

The patient was hypotensive on presentation to the emergency department with a blood pressure (BP) of 80/44 mmHg, heart rate of 124 beats/min, body temperature of 36.9°C, white cell count (WCC) of 14.26 × 10^9^/L, C‐reactive protein (CRP) of <5 mg/L, and hemoglobin (Hb) of 125 g/L.

## 3 INVESTIGATIONS

**FIGURE 1:**
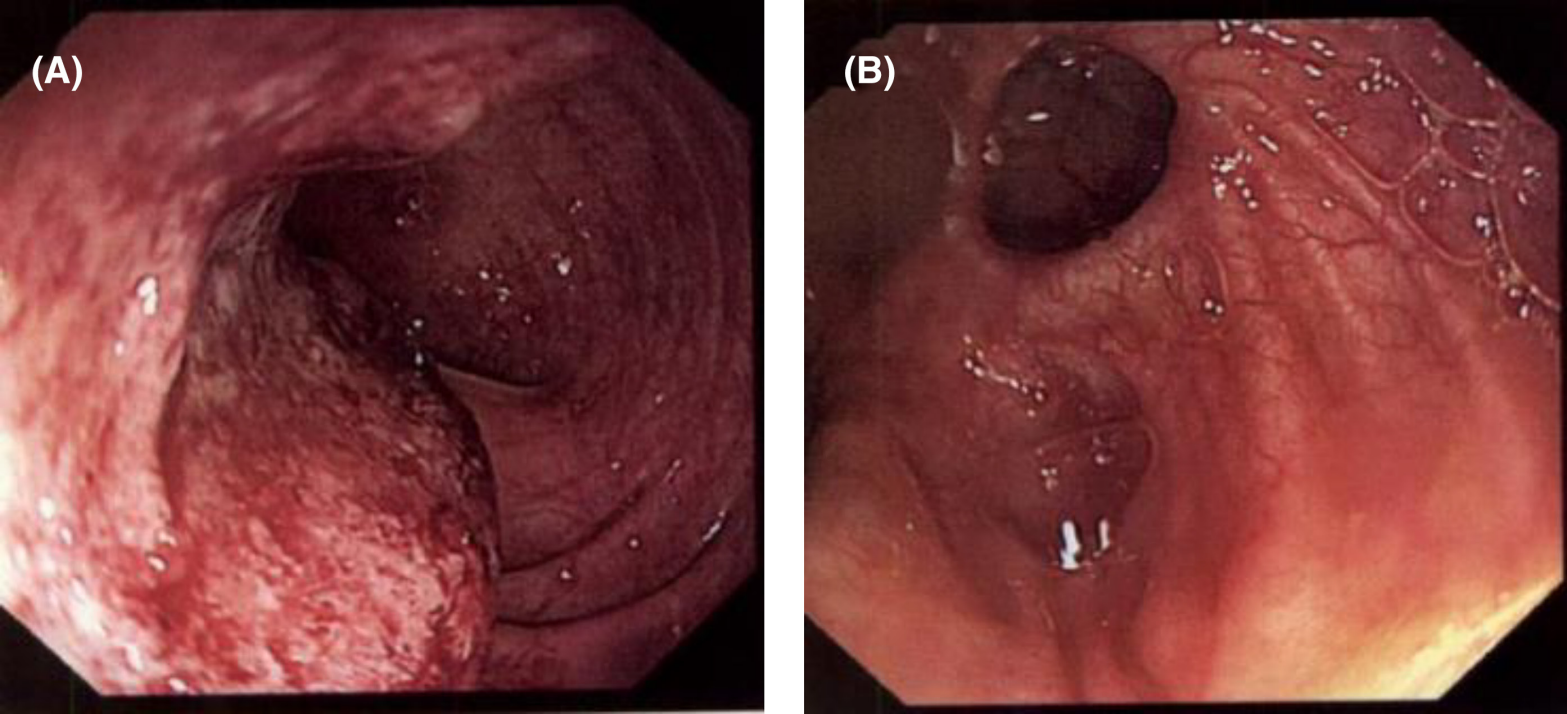
Colonoscopic findings. Colonoscopy showed: (A) 4‐cm malignant‐looking lesion in the sigmoid colon and (B) evidence of diverticular disease extending into the hepatic flexure.

A colonoscopy 3 weeks prior to admission, due to weight loss and diarrhea, showed a 4‐cm malignant‐looking lesion in the sigmoid colon, which was tattooed, and several diverticula, but no obvious fistulas were seen (Figure 1). Histopathological analysis of the sigmoid lesion showed high‐grade dysplasia.

**FIGURE 2:**
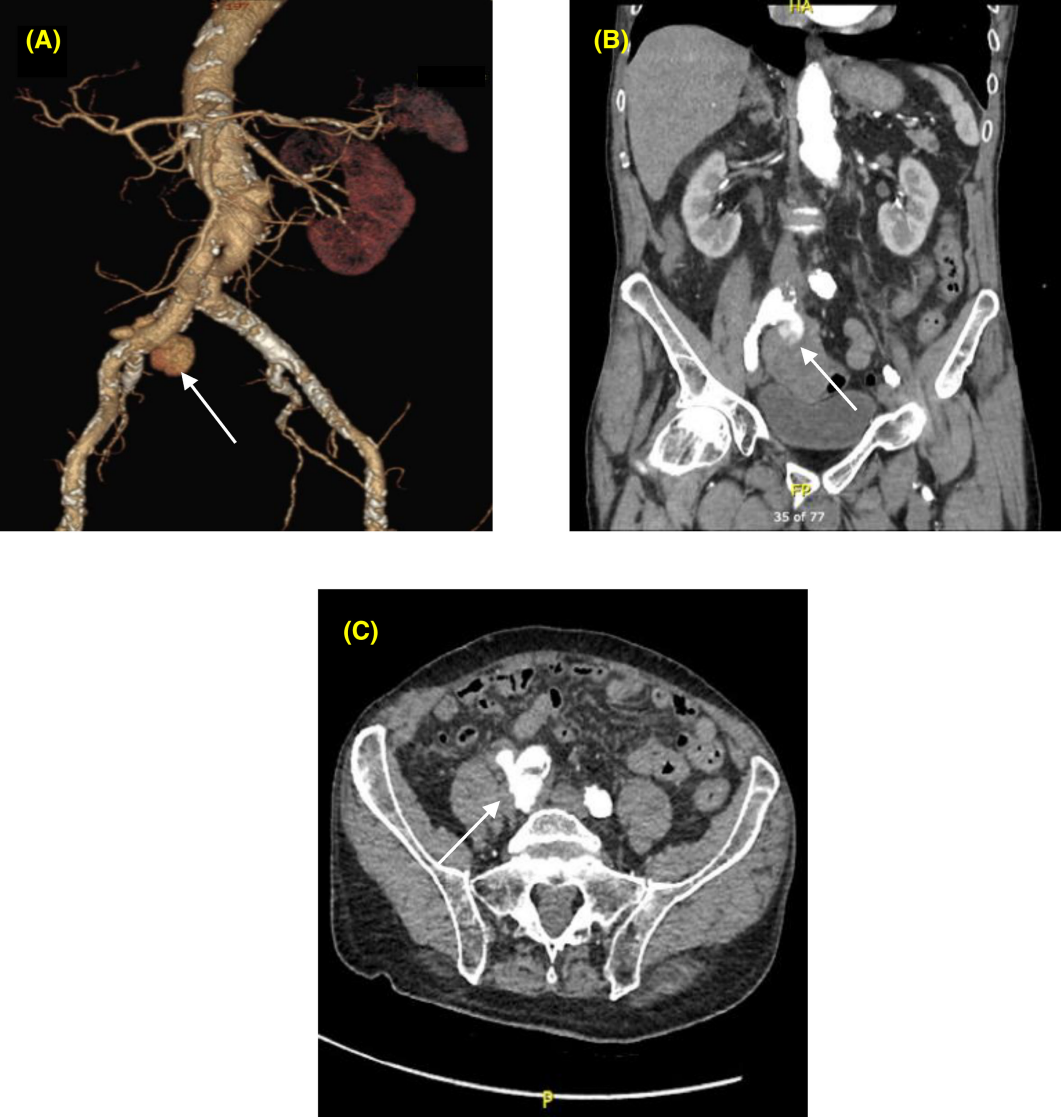
Computed tomography (CT) findings. (A) Digital subtraction CT angiography image demonstrating right common iliac artery anastomotic pseudoaneurysm (white arrow). (B) Coronal CT image demonstrating the site of fistulation into the sigmoid colon. (C) Axial image demonstrating the anatomy of the pseudoaneurysm.

An urgent triple‐phase computed tomography angiogram (CTA) showed a ruptured pseudoaneurysm at the anastomotic junction of right aortoiliac graft limb and common iliac artery, which appeared to fistula into the adjacent sigmoid colon. There was no obvious colonic metastatic disease (Figure 2A–C).

## 4 TREATMENT

The patient was fluid resuscitated according to our hospital's massive transfusion protocol of 3000 units of prothrombin complex concentrate (due to prolonged prothrombin time), 2 units of packed red cells, 1 unit of fresh frozen plasma, 1 g of tranexamic acid, and 10 mg of vitamin K. Prophylactic intravenous piperacillin–tazobactam was administered, given the likelihood of abdominal gut flora spreading onto the prosthetic material. His rivaroxaban and methotrexate were withheld.

Following initial stabilization steps and an urgent multidisciplinary team (MDT) meeting, it was decided that he would benefit from a temporizing endovascular procedure to prevent further exsanguination as a bridge to definitive surgical management.

**FIGURE 3:**
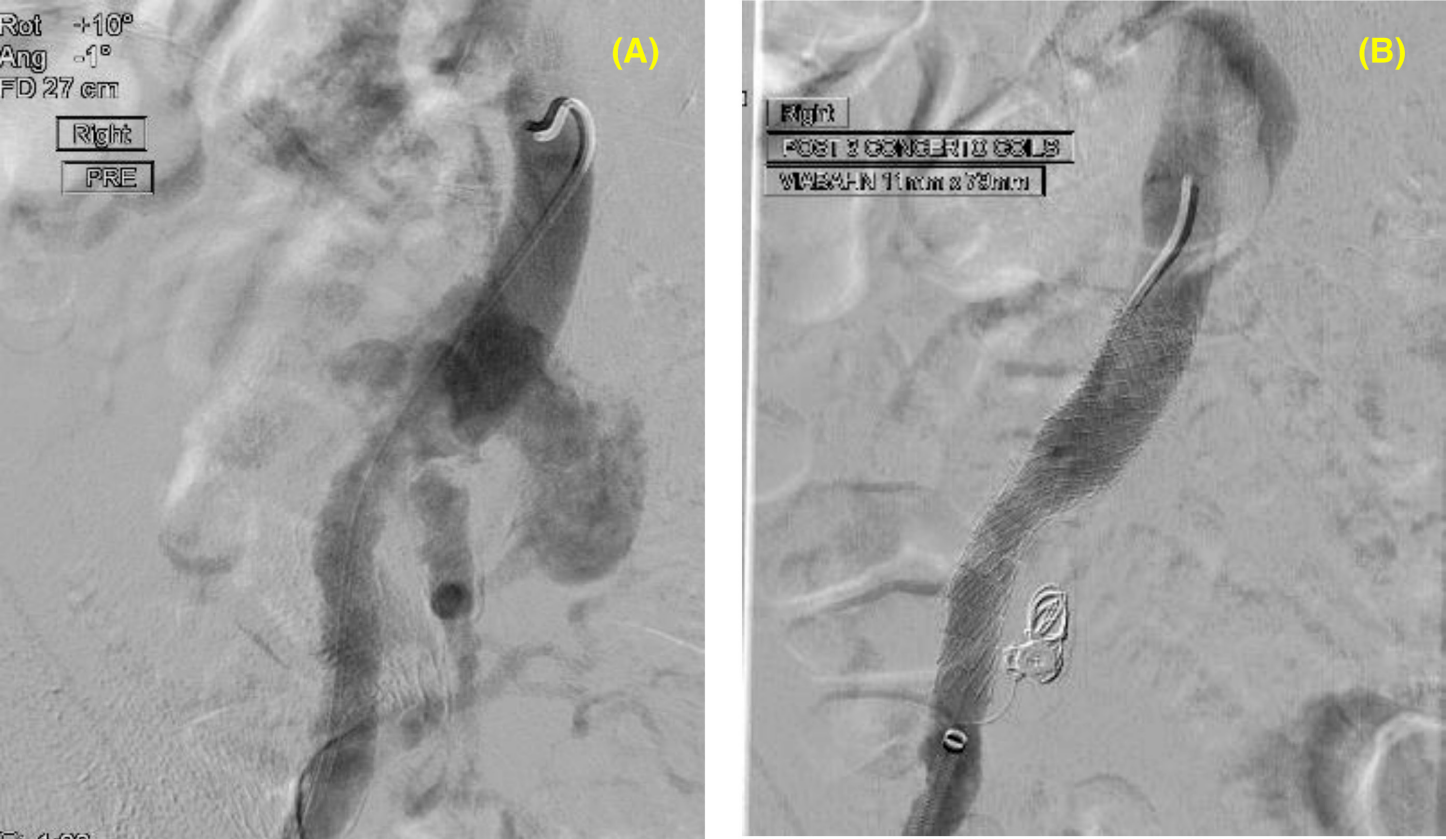
Angioplasty—image from angioplasty procedure demonstrating: (A) the pseudoaneurysm and (B) the exclusion of the pseudoaneurysm using an endoprosthesis (stent graft).

Ultrasound‐guided, percutaneous retrograde right common femoral artery access was secured, and an 8Fr sheath placed. The right internal iliac artery (IIA) was embolized with Concerto coils (Medtronic). A 11 × 79 mm balloon‐mounted endoprosthesis (Viabahn VBX, Gore®) was then deployed across the pseudoaneurysm, spanning from iliac limb graft into the external iliac artery (Figure 3). After a period of inhospital stabilization, the patient was discharged home as per his wishes over the Christmas period, with a view to convalescence and work up for definitive surgery. He was prescribed oral co‐amoxiclav 625 mg three times a day as a suppressive regime given the likely infected prosthetic material. His rivaroxaban and methotrexate were withheld.

Colorectal MDT gave a predictive staging of T4b, N0, and M0 sigmoid cancer, and the colorectal team advocated for an anterior resection with an end colostomy to mitigate any life‐threatening risks that might arise from an anastomotic leak. The vascular surgery team proposed explantation of the aortoiliac Y‐graft and reconstruction using autologous deep vein. Alternative “fallback” options were discussed, including full or partial explantation with extra‐anatomical bypass (femoro‐femoral or axillo‐femoral) or a more conservative approach with long‐term suppressive antibiotics.

Satisfactory performance on cardiopulmonary exercise testing (CPET) and echocardiography gave objective support to the patient's cardiorespiratory fitness to proceed with complex major abdominal surgery. Venous duplex ultrasound examination confirmed sufficient superficial femoral vein (SFV) as potential autologous conduit for arterial reconstruction. It was agreed that both the colorectal and vascular components of surgery should happen simultaneously.

Soon after the New Year, the patient was reviewed in the vascular surgery and colorectal outpatient clinic, where the proposed operative procedure, alternatives, risks, and benefits were explained to the patient and his wife. He expressed his wish to proceed, and a date for surgery was fixed.

The patient underwent a joint procedure between the colorectal and vascular teams, via midline laparotomy. The finding of a fistula between right iliac limb anastomosis and the sigmoid colon was confirmed. The colonic tattoo appeared remote from the area of corruption. There was macroscopic impression of entire aortic graft involvement in an infective/inflammatory process. The procedure entailed: (i) sigmoid colectomy with end colostomy; (ii) right SFV harvesting and fashioning of pantaloon bifurcated graft; (iii) AAA Y‐graft explantation, debridement of aortic sac, removal of Viabahn stent from CFA and arterial reconstruction with the autologous SFV graft, anastomosed to infrarenal aorta, the left CIA origins, and right EIA origin; and (iv) omental coverage of the graft via a fenestration in the transverse mesocolon. Microbiological specimens were taken, including the prosthetic graft, pseudoaneurysm sac and associated thrombus, and Viabahn stent graft.

There were no intraoperative complications during the procedure with an estimated blood loss of 2 L over the 9‐h operation. The patient was transferred to the intensive care unit (ICU) and extubated after 24 h. Microbiology results of the iliac stent graft demonstrated Citrobacter koseri, *E. coli*, Enterococcus faecium, and bacteroides. The aortic graft material showed evidence of *E. coli* and Enterococcus faecium. Based on antibiotic susceptibility, the patient was commenced on teicoplanin, ciprofloxacin, and metronidazole, which were continued for a 6‐week course.

## 5 OUTCOME AND FOLLOW‐UP

The patient stayed in the ICU for 4 days before being stepped down to the vascular ward for postoperative recovery totaling 30 days.

Postoperative complications included: (i) ileus requiring total parenteral nutrition for a period of 21 days, (ii) hospital acquired pneumonia, (iii) wound dehiscence of the midline laparotomy 9 days postoperatively requiring an emergency relook laparotomy and primary closure, (iv) right cerebellar embolic‐type infarction 25 days postoperatively; patient continued on rivaroxaban and did not require any further interventions given no focal neurological abnormalities, and (v) seroma in patient's right thigh secondary to vein harvesting which was treated conservatively with compression stocking.

Histopathology results of the sigmoid colectomy diagnosed a moderately differentiated adenocarcinoma stage IIA (pT3, pN0, pMx, R0) according to American Joint Committee on Cancer (AJCC) staging system,^9^ with clear resection margins. Interestingly, it also showed that diverticular disease was the most likely cause of the focal fistula and was therefore completely unrelated to the tumor.

The patient went on to make a full recovery and was discharged home to complete the antibiotic regime. His methotrexate was eventually changed to an IL‐23 inhibitor given the association between methotrexate and malignancy. On follow‐up at 3 and 6 months, the patient has made a full recovery and remains clinically well. Surveillance colonoscopy, serum carcinoembryonic antigen (CEA) levels, and CT scans were arranged as an outpatient according to NICE guidelines for follow‐up of colorectal cancer.

## 6 DISCUSSION

This case report is the first case in literature describing an iliac artery graft‐enteric fistula on the background of sigmoid cancer and diverticular disease. The acute endovascular “bridging” procedure followed by planned definitive surgical intervention proved to be successful in the management of such complex and potentially life‐threatening pathology.

Primary ArEFs are rarer than secondary ArEF with an incidence of only 0.04%–0.07% compared to the 0.36%–1.6% risk of developing a secondary ArEF after surgical treatment of aortic disease.^7^, ^11^, ^12^ Encountering iliac artery‐sigmoid fistulas in literature is rare; however, the majority involve patients with a history of atherosclerotic aneurysm, pelvic malignancy, or radiation.^8^, ^13^ An interesting feature of this particular case is the potential culprits that could have played a role in the development of a fistula, including diverticular disease, colorectal cancer, and a difficult colonoscopy.

It is the authors' hypothesis that the patient developed tethering of a diverticular segment of sigmoid colon onto the graft‐right CIA. Colonoscopic investigation of the red‐flag colonic cancer symptoms may have precipitated fistulation and pseudoaneurysmal degeneration, but this is difficult to be certain of and may have happened in any case.

Histopathological analysis provided evidence of a diverticula being directly involved in fistula formation and therefore was definitely a catalyst in the pathological process. Colorectal tumors are also associated with the development of fistulas,^14^ allowing us to assume that the adenocarcinoma may have influenced its formation via direct forces and inflammatory mechanisms. Colonoscopies do not have any direct correlating evidence in the pathophysiological development of ArEF; however, Khalaf et al.^15^ outlined a case report of a patient with an AEF into the sigmoid colon, which interestingly presented with lower GI bleeding during a colonoscopy procedure. In fact, colonoscopies have been associated with increased risk of rectocutaneous fistulas, which involves a similar pathophysiological process as ArEF.^16^

The presentation of ArEF is associated with a triad of gastrointestinal bleeding, abdominal pain, and a palpable abdominal mass; however, studies have shown that this is seen in only 11% of cases.^17^ Of clinical interest, an initial GI bleed followed by a massive or life‐threatening hemorrhage is typical of ArEF. The initial bleeding is likely to be self‐limiting due to vasospasm, thrombus formation, and hypotension.^17^ Permissive hypotension is a key in the management as excessive fluid replacement therapy may restore normotension leading to further bleeding and exsanguination.

Computed tomography angiogram is the gold standard for initial assessment with a reported sensitivity of 94% and specificity of 85% in recognizing ArEFs. The classical features to be seen on a CTA are: air within the arterial wall and extravasation of contrast into the gastrointestinal tract. Less common signs that may suggest the presence of ArEF are thickening of bowel wall in close proximity to an aneurysm or disrupted perivascular fat planes.^17^ Over the last few years, we have seen an emergence of endovascular techniques used for hemodynamic stabilization, control of sepsis, bridge to definitive surgical repair, and palliation for those at risk of major surgery. Danneel et al.^18^ have suggested using this “bridging” procedure for all those with IEF who are considered high‐risk for open repair. Endovascular repair may be considered as a short‐term management option to allow for a period of stabilization and planning for major complex definitive procedure to treat both the graft infection and the fistula.^18^ As a result, this time‐saving approach has allowed the patient to recover from the hemodynamic compromise, undergo preoperative investigations, and reduce risks from both local infection and systemic infection.^18^, ^19^, ^20^ Given the assumption that the aorto bi‐iliac graft was infected due to contact with the gut microbiota, the “gold standard” of complete explantation and reconstruction with autologous venous conduit was considered the preferred approach.^21^, ^22^ A two‐staged procedure, where the sigmoid cancer is removed first then followed by another procedure to treat the aneurysm, is arguably another strategy to treat the condition. We opted to perform simultaneous surgery for the following reasons: (i) one‐staged procedure was deemed safe considering the patient fitness and the availability of highly experienced colorectal and vascular surgeons; (ii) to avoid a second major laparotomy and its associated perioperative risks, and (iii) to prevent infective complications that may arise from an occult infection within the graft including risks of life‐threatening bleeding/rupture and thrombosis.

Graft infection can be asymptomatic with no clinical manifestations in afebrile patients who may show normal levels of infection markers.^18^, ^19^, ^20^ Peri‐ and postoperative bacterial suppression with prolonged course of antibiotics covering both gram‐positive and gram‐negative organisms is mandatory to reduce the risk of infective complications.

Numerous studies have found better outcomes when using autologous vein grafts rather than further synthetic graft material during reconstruction. Cryopreserved allografts, silver‐coated grafts, rifampicin‐bonded polyester grafts, or bovine pericardium are alternative options, but considered inferior to autologous deep vein in this case.^23^, ^24^, ^25^, ^26^, ^27^, ^28^, ^29^

## AUTHOR CONTRIBUTIONS

**Michael Azzopardi:** Data curation; formal analysis; writing – original draft. **Tom Wallace:** Supervision; visualization; writing – review and editing. **Yazan S. Khaled:** Conceptualization; formal analysis; investigation; supervision; visualization; writing – review and editing.

## CONFLICT OF INTEREST STATEMENT

The authors declare no conflict of interest.

## CONSENT STATEMENT

Written informed consent was obtained from the patient to publish this report in accordance with the journal's patient consent policy.

## Data Availability

Data sharing not applicable to this article as no datasets were generated or analysed during the current study.
